# Variation in the Outcome of Plant-Mediated Pathogen Interactions in Potato: Effects of Initial Infections on Conspecific vs. Heterospecific Subsequent Infections

**DOI:** 10.1007/s10886-023-01434-1

**Published:** 2023-05-19

**Authors:** Gabriela Quiroga, Naila Aguiño-Domínguez, Nikos Piperakis, Lucía Martín-Cacheda, Luis Abdala-Roberts, Xoaquín Moreira

**Affiliations:** 1 Misión Biológica de Galicia (MBG-CSIC), Apartado de correos 28, Pontevedra, Galicia, 36080 Spain; 2 Centro de Investigaciones Agrarias de Mabegondo (CIAM), Apartado de correos 10, Coruña, 15080 A Spain; 3 Faculty of Agriculture, Aristotle University of Thessaloniki, University Campus, Thessaloniki, 54124 Greece; 4 Departamento de Ecología Tropical, Campus de Ciencias Biológicas y Agropecuarias, Universidad Autónoma de Yucatán, Merida, Apartado Postal 4-116, Itzimná. 97000. Mérida, Yucatán México

**Keywords:** Plant-mediated interactions, sequential attack, *Alternaria solani*, *Phytophthora infestans*, *Solanum tuberosum*, plant defences

## Abstract

Plants are often attacked sequentially by multiple enemies. Pathogen sequential co-infections can lead to indirect interactions mediated by plant induced responses whose outcome is contingent on differences in the magnitude and type of plant induced defences elicited by different species or guilds. To date, however, most studies have tested unidirectional effects of one pathogen on another, not discerning between conspecific vs. heterospecific infections, and often not measuring plant induced responses underlying such outcomes. To address this, we conducted a greenhouse experiment testing for the impact of initial infection by two leaf pathogens (*Alternaria solani* and *Phytophthora infestans*) on subsequent infection by each of these pathogens on potato (*Solanum tuberosum*) plants, and also measured induced plant defences (phenolic compounds) to inform on interaction outcomes. We found contrasting results depending on the identity of the initially infecting pathogen. Specifically, initial infection by *A. solani* drove induced resistance (lower necrosis) by subsequently infecting *A. solani* (conspecific induced resistance) but had no effect on subsequent infection by *P. infestans*. In contrast, initial infection by *P. infestans* drove induced resistance to subsequent infection by both conspecifics and *A. solani*. Patterns of plant induced defences correlated with (and potentially explained) induced resistance to conspecific but not heterospecific (e.g., in the case of *P. infestans*) subsequent infection. Overall, these results further our understanding of plant-mediated pathogen interactions by showing that plant-mediated interactions between pathogen species can be asymmetrical and in some cases not reciprocal, that pathogen species can vary in the importance of conspecific vs. heterospecific effects, and shed mechanistic insight into the role of plant induced responses driving such interactions.

## Introduction

Plants are constantly under attack by above- and below-ground enemies such as viruses, bacteria, fungi, nematodes and insect herbivores (Stout et al. 2006). The effects of these attackers can take place either simultaneously or sequentially, and in the latter case lead to plant-mediated indirect interactions where early-arriving attackers positively or negatively affect the performance of subsequent attackers (Wang et al. 2014; Moreira et al. 2015; Biru et al. 2022). These plant‐mediated interactions are determined by the magnitude as well as type of plant responses which involve both mechanical (e.g. spines, trichomes) and chemical (e.g. secondary metabolites) defensive traits (War et al. 2012).

Research on plant induced responses has shown that attack activates one or more hormonal signalling pathways associated with induction of plant defences (e.g. salicylic acid and jasmonic acid pathways) (Pieterse et al. 2012; Erb et al. 2012). The activation of a given pathway is frequently enemy-specific and in some cases can be predicted based on the enemy’s feeding mode or lifestyle (e.g. guild or functional group; Thaler et al. 2012). Enemies of the same feeding guild are expected to activate the same pathway whereas different guilds should activate different pathways and this specificity informs the outcome of sequential interactions by the same or different plant enemies (Thaler et al. 2012). For example, the cross-talk hypothesis predicts that initial attackers inducing defences through a given pathway should have negative effects on subsequent attackers (conspecifics or heterospecifics) inducing the same pathway (i.e. induced resistance) but positive effects on subsequent attackers inducing a different pathway (induced susceptibility), in the latter case due to interference between pathways (Pieterse et al. 2006, Stout et al. 2006, Thaler et al. 2012).

Although there is good evidence for interactions between plant defensive pathways (e.g. crosstalk) for some plant species (reviewed by Thaler et al. 2012), in other cases results have been inconsistent or unsupportive (Schenk et al. 2000; van Wees et al. 2000; Tsuda et al. 2009). Indeed, attackers within the same guild sometimes exhibit variation in their effects on plant pathways and hormonal pathways are highly reticulate and complex which in some cases makes it difficult to predict levels of defensive end products and thus interaction outcomes based on species identity or guild (Fernandez-Conradi et al. 2018; Moreira et al. 2018). In the case of plant pathogens, this appears to be especially the case and a recent meta-analysis by Moreira et al. (2018) reported limited evidence for the predictability and occurrence of indirect interactions based on the identity of pathogen guilds. Nonetheless, the number of studies with pathogens remains low and results across studies are highly variable (see Table 1), pointing the need for more studies. In doing so, two key, but often neglected features emerge. One is the importance of testing for reciprocal effects between pathogens (rather than unidirectional effects) and the other is to measure the plant induced responses. Addressing these aspects would allow to test for initial pathogen effects on conspecific vs. heterospecific subsequent pathogens, as well as reveal plant induced responses underlying interaction outcomes.

**Table 1:** Summary of the published studies investigating the effects of initial pathogen infection on subsequent pathogen infection. We describe the type of initial and subsequent pathogens (e.g. nematode, biotrophic or necrotrophic fungus, bacteria or oomycete) and the hormonal signalling pathway induced by pathogens (JA = jasmonic acid, SA = salicylic acid)

Study	Initial attacker	Enemy type	Subsequent attacker	Enemy type	Pathway	Response
Russin et al. 1989	*Heterodera glycines*	Nematode	*Diaporthe phaseolorum caulivora*	Necrotrophic fungus	JA_JA	Induced systemic resistance
Simon and Hilker 2003	*Melampsora allii-fragilis*	Biotrophic fungus	*Melampsora allii-fragilis*	Biotrophic fungus	SA_SA	Induced systemic susceptibility
Cui et al. 2005	*Pseudomonas syringae*	Hemibiotrophic bacteria	*Pseudomonas syringae*	Hemibiotrophic bacteria	SA_SA	Induced systemic susceptibility
Blodgett et al. 2007	* S. sapinea/Diplodia scrobiculata*	Necrotrophic fungus	*Sphaeropsis sapinea*	Necrotrophic fungus	JA_JA	Organ-dependent induced systemic resistance/susceptibility
Eyles et al. 2007	*Sphaeropsis sapinea*	Necrotrophic fungus	*Sphaeropsis sapinea*	Necrotrophic fungus	JA_JA	Induced systemic resistance
Spoel et al. 2007	*Pseudomonas syringae*	Hemibiotrophic bacteria	*Alternaria brassicicola*	Necrotrophic fungus	SA_JA	Induced local susceptibility
Laine 2011	*Podosphaera plantaginis*	Biotrophic fungus	*Podosphaera plantaginis*	Biotrophic fungus	SA_SA	Induced systemic susceptibility (field)/ resistance (laboratory)
Vos et al. 2015	*Hyaloperonospora arabidopsidis*	Biotrophic oomycete	*Botrytis cinerea*	Necrotrophic fungus	SA_JA	Induced systemic susceptibility
Moreira et al. 2021	*Sclerotinia sclerotiorum*	Necrotrophic fungus	*Sclerotinia sclerotiorum*	Necrotrophic fungus	JA_JA	Induced systemic resistance

In this study, we tested for effects of initial infection by two foliar pathogens (*Alternaria solani* and *Phytophthora infestans*) on subsequent infection by each other on potato (*Solanum tuberosum*) plants. In addition, we further measured induced plant defences (phenolic compounds) potentially underlying the effects of initial infection on subsequent infection. Specifically, we asked the following: (1) Are there plant-mediated pathogen effects and are interaction outcomes contingent on the identity of the initially infecting pathogen? We were interested in comparing initial pathogen effects on conspecific vs. heterospecific subsequent pathogens and whether pathogen species affected each other reciprocally. (2) Do pathogens induce plant defences (phenolic compounds), do such responses vary depending on the identity of infecting pathogens, and do induced defences correlate with (and thus potentially explain) interaction outcomes? The selected fungi cause common and severe potato diseases worldwide and have different infection strategies. *Alternaria solani* is a specialist necrotrophic fungus inducing the jasmonic acid pathway (Thomma 2003), whereas *P. infestans* is a generalist hemibiotrophic oomycete inducing the salicylic acid pathway (Zuluaga et al. 2015). In addition, the chosen plant defences, phenolics (e.g. hydroxycinnamic acids, flavonoids, tannins) constitute one the more widespread group of defensive compounds, acting against both herbivores and pathogens (Wallis and Galarneau 2020), and are induced by both of the studied pathogens (Andreu et al. 2001; Flors et al. 2003; Mittelstraß et al. 2006). Overall, this study aims to provide a better understanding of plant-mediated pathogen interactions, and explores the role of plant induced responses in driving such interactions.

## Methods and Materials

*Study System. Solanum tuberosum* L. (*Solanaceae*) is a plant that grows up to 60 cm high and propagates from seeds and tubers. It was introduced in Europe during the second half of the 16th century from the Central Andes region (Peru-Bolivia) where it was domesticated between 8,000 and 10,000 years ago (Pearsall 2008). It is the third most-produced crop following rice and wheat and the first non-cereal crop in the world with an annual production of over 380 million tons (FAOSTAT, 2020).

Agricultural intensification, added to the acute climate change crisis, hinders the control of crop pests and diseases, compromising food safety. In particular, potato is susceptible to a wide range of plant pathogens which cause severe quality and yield losses (Birgit et al. 2020; Devaux et al. 2020). Amongst these, the two most important pathogens are late blight (*Phytophthora infestans*) and early blight (*Alternaria solani*) (Vilvert et al. 2022). *Phytophthora infestans* is a generalist hemibiotrophic oomycete (Zuluaga et al. 2015), that produces brown or black spots on the surface of potato leaves and stems. *Alternaria solani* is a specialist necrotrophic fungus whose main symptoms are also dark brown to black necrosis in leaves (Thomma 2003). Infection by these pathogens causes the decay of infected tissues, eventually leading to plant death (Birgit et al. 2020).

*Experimental Design.* In July 2021, we individually planted tubers of potato variety *S. tuberosum* L. cv. Desiree in 4 l pots containing potting soil and peat (Gramoflor GmbH & Co. KG Produktion, Vechta, Germany). Plants were grown in a glasshouse under controlled light (minimum 10 h per day, Photosynthetically Active Radiation = 725 ± 19 µmol m-2 s^− 1^) and temperature (10 °C night, 25 °C day), and were watered twice a week to field capacity.

Three weeks after tuber planting, when plants were 31.78 ± 0.52 cm in height, we randomly assigned plants to one of three treatments: (1) *P. infestans* infection, (2) *A. solani* infection or (3) control (intact plants). For the infection treatments, we applied three punctures on the upper side of one fully developed leaf using an awl of 1 mm of diameter, and then added agar plugs (0.4 cm in diameter) containing pathogen mycelia on the punctured site (Moreira et al. 2021). For the control treatment, we punctured the leaves as above to control for damage due to puncturing but did not add the pathogen-containing agar. One week after initial treatment establishment, we randomly selected half of the plants of each group to one of the following subsequent infection treatments: *A. solani* or *P. infestans* infection. In this case, we followed the same procedure as above, using one a fully developed leaf on the opposite branch to the leaf used for initial infection. In total, the experiment included 150 plants, corresponding to three initial infection treatments × two subsequent infection treatments × 25 replicates per combination. One week after the second infection, we collected leaves with symptoms of necrosis and photographed them with a Nikon COOLPIX P100 digital camera (10.3 effective megapixels, 26× zoom NIKKOR). We estimated the percentage of necrotic area (hereafter leaf necrosis) due to pathogen infection using ImageJ software (version 1.52a; LOCI, University of Wisconsin, USA).

In addition, to test for variation in pathogen effects on plant induced defences possibly explaining interaction outcomes, one week after the second infection (at the same time as necrosis measurements) we collected three fully expanded (uninfected) leaves located on the same branch as leaves infected by the second pathogen. We then oven-dried these leaves for 48 h at 40 °C and ground them to quantify phenolic compounds (see ahead). Sampling leaves after the second infection addresses the compounded effects of both infections on induced defences and provides a measure of induced levels during ongoing infection by the subsequent pathogen which could inform interaction outcomes.

*Chemical Analyses.* We extracted phenolic compounds from 20 mg of dry leaf tissue with 1 mL of 70% methanol in an ultrasonic bath for 15 min, followed by centrifugation (Moreira et al. 2014). We then transferred the extracts to chromatographic vials. For phenolic compound identification, we used an ultra-performance liquid chromatography coupled with electrospray ionization quadrupole (Thermo Dionex Ultimate 3000 LC) time-of-flight mass spectrometry (UPLC-Q-TOF-MS/MS) (Bruker Compact™). We performed chromatographic separation in a Kinetex™ 2.6 μm C18 82–102 Å, LC Column 100 × 4.6 mm column using a binary gradient solvent mode consisting of 0.1% formic acid in water (solvent A) and acetonitrile (solvent B). We used the following gradient: from 5 to 30% B (0–6 min), from 30 to 60% B (6–14 min), from 60 to 80% B (14–17 min), from 80 to 100%B (17–19 min), from 100 to 5% B (19–24 min). The injection volume was 5µL, the flow rate was established at 0.4 ml/min and column temperature was controlled at 25 °C. MS analysis was operated in a spectra acquisition range from 50 to 1200 m/z. Negative (-) ESI modes was used under the following specific conditions: gas flow 8 l/min, nebulizer pressure 38 psi, dry gas 7 l/min, and dry temperature 220 °C. Capillary and end plate offset were set to 4500 and 500 V, respectively. MS/MS analysis was performed based on the previously determined accurate mass and RT and fragmented by using different collision energy ramps to cover a range from 15 to 50 eV. Individual compounds were identified based on the data obtained from the standard substances including RT, λmax, ([M–H]−), and major fragment ions. For phenolic compound quantification, 5 µL of each sample was then analysed using the same column and conditions mentioned in the previous paragraph, in an UHPLC (Nexera LC-30AD; Shimadzu) equipped with a Nexera SIL-30AC injector and one SPD-M20A UV/VIS photodiode array detector. Chromatograms were recorded at 330 nm. We identified two groups of phenolic compounds, namely flavonoids (one unidentified compound) and hydroxycinnamic acids (three compounds: 1-caffeoyl glucose, 3-caffeoyl quinic acid, 5-caffeoyl derivative). We quantified flavonoids as rutin equivalents, and hydroxycinnamic acids as ferulic acid equivalents. We achieved the quantification of these phenolic compounds by external calibration using calibration curves at 0.25, 0.5, 1, 2 and 5 µg mL^− 1^ for each standard. We calculated total phenolics as the sum of flavonoids and hydroxycinnamic acids, and expressed concentrations in mg g^− 1^ tissue on a dry weight basis.

*Statistical Analyses.* Firstly, we tested the effects of each initially infecting pathogen (*A. solani* or *P. infestans*) on leaf necrosis by running a general linear model testing for the effect of pathogen infection on leaf necrosis. This initial analysis resulted in non-significant differences between the two pathogens on leaf necrosis (DF = 1, 97; t-ratio= -0.655; *P* = 0.5137). Secondly, we tested the effects of each initially infecting pathogen separately on leaf necrosis and plant chemical defences (hydroxycinnamic acids and flavonoids) by using different (non-mutually exclusive, see below) subsets of the full data set. In the case of the *A. solani* initial infection, we included control plants subsequently infected by *A. solani* (A), plants initially and subsequently infected by *A. solani* (B), controls subsequently infected by *P. infestans* (C), and plants initially infected by *A. solani* and subsequently infected by *P. infestans* (D). Likewise, for the *P. infestans* initial infection we included control plants subsequently infected by *P. infestans* (A), plants initially and subsequently infected by *P. infestans* (B), controls subsequently infected by *A. solani* (C), and plants initially infected by *P. infestans* and subsequently infected by *A. solani*. In each case, we ran a general linear model testing for the effect of sequence of infection, coding the above treatment combinations as levels of this factor. We then performed pre-planned contrasts testing for differences between A vs. B and C vs. D, i.e. conspecific and heterospecific effects, respectively. Reciprocal effects between pathogens were assessed qualitatively by comparing initial pathogen effects on the heterospecific pathogen, i.e. whether effects were significant in one or both cases, and the direction of any such effects. Similarly, we ran analyses for leaf hydroxycinnamic acids and flavonoids following the same model structure and contrasts. We log-transformed leaf necrosis, hydroxycinnamic acids and flavonoids to achieve normality of residuals.

We ran all statistical analyses in R software version 4.1.1 (R Core Team 2018). We implemented linear models using the *lmer* function from the *lmerTest* package (Kuznetsova et al. 2017). We obtained least-squared means, standard errors and post hoc Tukey comparisons from these models using the *lsmeans* function from the *lsmeans* package (Lenth 2016).

## Results

**Fig. 1:**
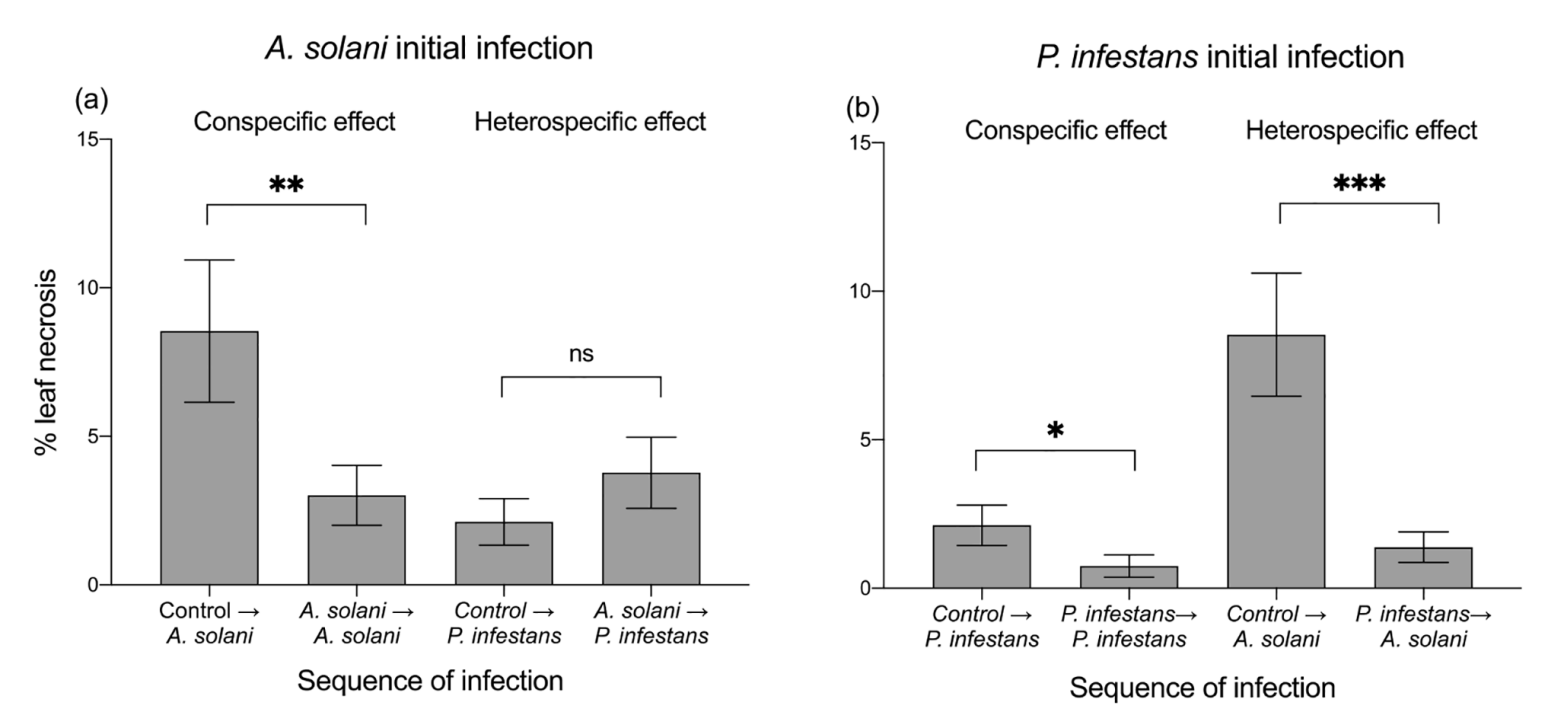
Percentage of leaf necrotic area caused by a subsequent infection by (a) *Alternaria solani* or (b) *Phytophthora infestans* after initial infection (two levels: control and infected by *A. solani* or *P. infestans*) in potato (*Solanum tuberosum*) plants. For each pathogen, we compared subsequent infection by a given pathogen on initial controls vs. subsequent infection by that pathogen infection with initial infection by the same pathogen (i.e. conspecific induced resistance) and vs. subsequent infection by that pathogen with initial infection by the other pathogen (i.e. heterospecific induced resistance). Bars are least square means ± standard error (N = 25). Asterisks indicate significant differences within treatments at *P* < 0.05 based on Tukey post hoc tests. Results are shown in Table 2

*Effects of Initial Infection on Subsequent Infections.* Sequence of infection significantly affected percent necrosis by subsequent pathogens (Table 2), but we found variable outcomes depending on the identity of the initially infecting pathogen. Specifically, plants first infected by *A. solani* caused a significant 65% reduction in leaf necrosis after subsequent conspecific infections by *A. solani* relative to controls subsequently infected by this pathogen (i.e. conspecific induced resistance) (DF = 1,48; t ratio=-3.367, *P* = 0.002), but had no effect on leaf necrosis after subsequent infection by *P. infestans* compared to controls subsequently infected by this pathogen (DF = 1,48; t ratio = 0.996, *P* = 0.324) (Fig. 1a). In contrast, plants initially infected by *P. infestans* caused significant 65% and 84% reductions (respectively) in leaf necrosis in subsequent infections by both *P. infestans* and *A. solani* (i.e. conspecific and heterospecific induced resistance) relative to controls subsequently infected by the corresponding pathogen in each case (DF = 1,47; t ratio = 2.350, *P* = 0.021 and DF = 1,46; t ratio = 4.343, *P* < 0.0001) (Fig. 1b).

**Table 2:** Effects of sequence of infection on percent necrosis by subsequent pathogens and foliar concentration of flavonoids and hydroxycinnamic acids. In the case of the *A. solani* initial infection, we included control plants subsequently infected by *A. solani* (A), plants initially and subsequently infected by *A. solani* (B), controls subsequently infected by *P. infestans* (C), and plants initially infected by *A. solani* and subsequently infected by *P. infestans* (D). Likewise, for the *P. infestans* initial infection we included control plants subsequently infected by *P. infestans* (A), plants initially and subsequently infected by *P. infestans* (B), controls subsequently infected by *A. solani* (C), and plants initially infected by *P. infestans* and subsequently infected by *A. solani*. For each initial pathogen, we ran a general linear model testing for the effect of sequence of infection, coding the above treatment combinations as a level within this factor. We then performed pre-planned contrasts testing for differences between A vs. B and C vs. D, i.e. conspecific and heterospecific effects, respectively. F-values with degrees of freedom (numerator, denominator) and associated significance levels (*P*-values) are shown. Significant *P*-values (*P* < 0.05) are in bold

	Percent necrosis		Flavonoids		Hydroxycinnamic acids	
Sequence of arrival	F_3,94_	*P*	F_3,94_	*P*	F_3,94_	*P*
*A. solani initial infection* (T)	3.71	**0.014**	3.898	**0.011**	2.19	0.095
*P. infestans initial infection*	11.39	**< 0.001**	5.100	**0.003**	2.23	0.090

**Fig. 2:**
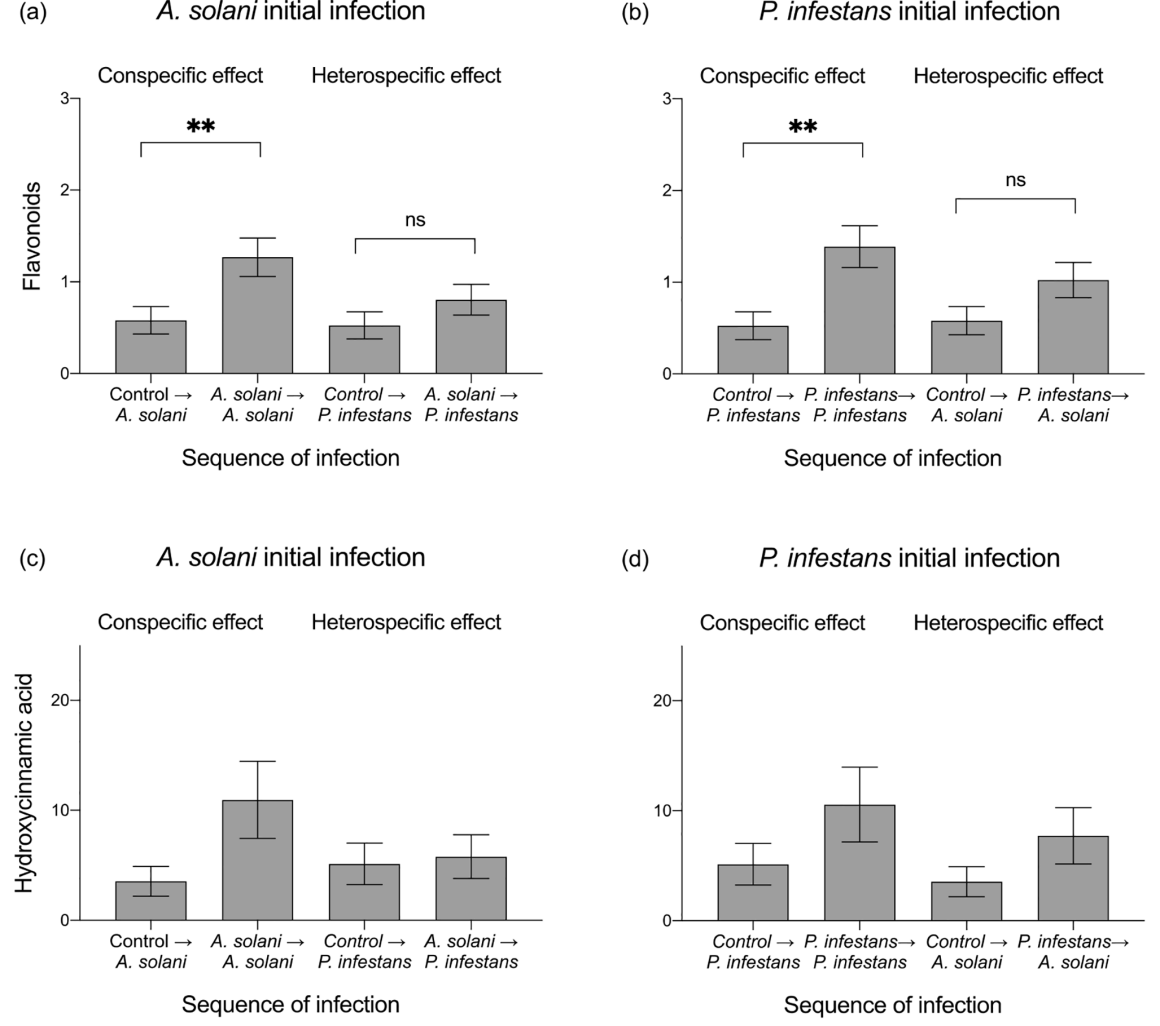
Concentration (in mg g^− 1^ d.w.) of flavonoids and hydroxycinnamic acids after initial infection (two levels: control and infected by *A. solani* or *P. infestans*) in potato (*Solanum tuberosum*) plants. For each pathogen, we compared subsequent infection by a given pathogen on initial controls vs. subsequent infection by that pathogen infection with initial infection by the same pathogen (i.e. conspecific induced resistance) and vs. subsequent infection by that pathogen with initial infection by the other pathogen (i.e. heterospecific induced resistance). Bars are least square means ± standard error (N = 25). Asterisks indicate significant differences within treatments at *P* < 0.05 based on Tukey post hoc tests. Results are shown in Table 2

*Effects of Pathogen Infections on Induced Plant Defences.* Sequence of infection significantly affected the concentration of leaf flavonoids (Table 2), but outcomes depended on the identity of the initial pathogen. Namely, flavonoid concentration was significantly (2.2-fold) higher after initial plus subsequent infections by *A. solani* relative to controls subsequently infected by this pathogen (DF = 1,47; t ratio = 2.657, *P* = 0.011) but did not differ significantly after initial infection by *A. solani* and subsequent infection by *P. infestans* relative to controls subsequently infected by *P. infestans* (DF = 1,46; t ratio = 1.308, *P* = 0.197) (Fig. 2a). Similarly, there was 2.6-fold significant increase flavonoids after initial and subsequent infections by *P. infestans* relative to controls subsequently infected by this pathogen (DF = 1,45; t ratio=-3.660, *P* = 0.001), but no difference after initial infection by *P. infestans* and subsequent by *A. solani* compared to controls subsequently infected by *A. solani* (DF = 1,47; t ratio=-1.447, *P* = 0.155) (Fig. 2b). There was no significant effect of sequence of infection on the concentration of hydroxycinnamic (Table 2), though trends, particularly for conspecific effects (Fig. 2c, d), were similar to flavonoids.

## Discussion

Initial infection by both pathogens drove induced resistance (i.e. reduction in leaf necrosis) to subsequent conspecific infections in potato plants. In contrast, effects of initial infection on subsequent heterospecific infections were contingent on the identity of the initial pathogen. Namely, *P. infestans* initial infection drove induced resistance to subsequent *A. solani* whereas initial infection by *A. solani* had no detectable effect on subsequent infection by *P. infestans*. In addition, patterns of plant induced defences were consistent with, and possibly explained, induced resistance to conspecific subsequent infection for both pathogens but not with effects on heterospecific subsequent infection. Together, these results indicate variable outcomes in plant-mediated pathogen interactions, namely in conspecific vs. heterospecific effects and a lack of reciprocity between pathogens, suggesting ample opportunity for priority effects to guide contrasting outcomes in plant-mediated pathogen interactions and their effects on plant performance.

Our results showed that initial infection by the necrotrophic pathogen *A. solani* triggered induced resistance against subsequently infecting conspecifics. In the case of potato, a recent study by our group similarly found that initial infection by the necrotrophic fungus *Sclerotinia sclerotiorum* significantly reduced subsequent infection by this pathogen (Moreira et al. 2021). Evidence of conspecific induced resistance involving necrotrophic pathogens has also been reported in other systems. For example, Eyles et al. (2007) found that initial infection by the necrotrophic fungal pathogen *Sphaeropsis sapinea* in pine trees reduced subsequent infection by this pathogen (see other studies in Table 1). In addition, our results showed that initial infection by the biotrophic pathogen *P. infestans* also reduced subsequent infection by this pathogen. In this case, however, evidence from other systems for conspecific induced resistance in SA-inducing biotrophic pathogens is less strong and cases of induced susceptibility are not uncommon (see Table 1). For instance, initial infection by the biotrophic fungal pathogen *Melampsora allii-fragilis* in willow trees (Simon and Hilker 2003) and the hemibiotrophic bacterium *Pseudomonas syringae* in *Arabidopsis thaliana* (Cui et al. 2005) each caused induced susceptibility to subsequent infection by conspecific pathogens. Recent work has shown that some biotrophic pathogens associated with the SA pathway can induce low to moderate amounts of JA with this in turn inhibiting the expression of genes associated with SA-related defences (Biere and Goverse 2016), possibly explaining the absence of effects of SA-inducing pathogens on subsequent conspecific infections. Accordingly, a recent meta-analysis found that JA-related pathogens had on average negative effects on subsequently infecting conspecifics whereas SA-related effects were more variable and non-significant overall (Moreira et al. 2018). Regardless of these findings, the fact that P. infestans drove induced resistance against subsequently infecting conspecifics by *P. infestans* suggests that interference between pathways due to dual SA-JA induction did not take place or was not strong enough to affect these interaction outcomes.

Several studies have reported that initially attacking plant enemies affect the performance of subsequent heterospecific attackers, mainly for insect herbivores (reviewed by Pieterse et al. 2006, Stout et al. 2006, Thaler et al. 2012), and to a lesser extent pathogens (reviewed by Moreira et al. 2018). Results from our study were unsupportive of plant defensive crosstalk which predicts initial attackers cause plant induced susceptibility to subsequent pathogens associated to a different pathway (i.e. positive indirect effect). Instead, we found that initial infection by *P. infestans*, a SA-associated biotroph, drove induced resistance to subsequently infecting *A. solani*, a JA-associated necrotroph, whereas initial infection by latter had no detectable effect on subsequent infection by *P. infestans*. These results are not consistent with patterns from our meta-analysis which showed that SA-related pathogens had no overall effect on subsequent JA-related pathogens, whereas JA-related pathogens had a negative effect on subsequently infecting SA-related pathogens (Moreira et al. 2018). Necrotrophs such as *A. solani* have been found to disrupt plant defence signalling (Prins et al. 2000; Sharon et al. 2007) for detoxification of host metabolites that interfere with virulence (Morrissey and Osbourn 1999), which could weaken or prevent effects of initial necrotrophic pathogens on subsequent pathogens. The fact *P. infestans* drove induced resistance to *A. solani* can be explained by the type of plant induced response triggered by the former which involves the synthesis of a broad array of antimicrobial compounds, including flavonoids, coumarins, and lignans through the phenylpropanoid pathway (Fraser et al. 2011; Lopes-Martin et al. 2020), potentially affecting a both necrotrophic and biotrophic pathogens. It is also possible that the observed outcome was specific to the pathogen studied, whereby *P. infestans* happens to induce specific types of plant defences that confer resistance against *A. solani*. Follow-up work testing effects of *P. infestans* infection on other pathogens associated to different pathways and susceptible to different groups of secondary metabolites is still missing. More broadly, studies are needed which test for reciprocal effects of biotrophic and necrotrophic pathogens to reach some degree of generalization on the occurrence, strength and direction of plant-mediated effects and pathogen (and plant) traits driving such outcomes.

In the case of plant induced defences, results indicated for both pathogens that levels of phenolic compounds (mainly flavonoids) significantly increased after initial and subsequent conspecific infections (relative to controls exposed to a single infection by the corresponding pathogen), possibly explaining induced resistance to conspecific subsequent infections for both pathogens. Previous studies in tomato(González-García et al. 2021) and potato (Tsypurskaya et al. 2022) showed that flavonoids conferred resistance against both pathogens, consistent with the idea that flavonoid induction mediated induced resistance to subsequently infecting conspecific pathogens. However, in the case of heterospecific infections, for both pathogens we found that flavonoid levels after initial and heterospecific subsequent infection were not significantly higher than for controls exposed to a single infection by the corresponding subsequent pathogen. In the case of *P. infestans*, and in contrast with consistent patterns of induced resistance to conspecific subsequent infection and plant defence induction by this pathogen, such findings suggest that the phenolic groups studied were not associated (at least not detectably) with the heterospecific effect of *P. infestans* on subsequent infection by *A. solani*. By the same token, it also suggests effects of *P. infestans* on conspecific vs. heterospecific subsequent infections were driven by different types of induced compounds (including unmeasured groups) or by qualitative differences in induced responses for the studied compound groups (specific compounds or changes in compound composition; Mittelstraß et al. 2006). In addition, for *A. solani*, the fact that this pathogen did not affect subsequent *P. infestans* infection, but showed consistent patterns of induced resistance to conspecific subsequent infection and plant defence induction, suggests that the measured induced defences (and/or other unmeasured compounds) provide resistance against conspecific subsequent infection but not *P. infestans*. We cannot discard either the possibility that *A. solani*, as other necrotrophs, inhibited the induction of unmeasured compounds with strong effects on *P. infestans* (see above), this weakening the effect on the latter. Chemical analyses that encompass other classes of compounds and with greater resolution to measure patterns of induction for specific compounds within groups are needed to establish a more robust link between induced changes in chemical defences and the outcome of pathogen interactions in potato and other systems as well.

In summary, our results further our understanding of plant-mediated pathogen effects by revealing interactions asymmetries, namely that effects between pathogen species may not be reciprocal and that pathogen species can vary in the importance of conspecific vs. heterospecific effects. Further, results also shed insight on underlying plant induced responses involving phenolic compounds and their role in shaping interaction outcomes.
